# Distinct characteristics of the gut virome in patients with osteoarthritis and gouty arthritis

**DOI:** 10.1186/s12967-024-05374-6

**Published:** 2024-06-13

**Authors:** Chang-Ming Chen, Qiu-Long Yan, Ruo-Chun Guo, Fang Tang, Min-Hui Wang, Han-Zhi Yi, Chun-Xia Huang, Can Liu, Qiu-Yi Wang, Wei-Ya Lan, Zong Jiang, Yu-Zheng Yang, Guang-Yang Wang, Ai-Qin Zhang, Jie Ma, Yan Zhang, Wei You, Hayan Ullah, Yue Zhang, Sheng-Hui Li, Xue-Ming Yao, Wen Sun, Wu-Kai Ma

**Affiliations:** 1 Department of Rheumatology and Immunology, The Second Affiliated Hospital of Guizhou University of Traditional Chinese Medicine, Guiyang, China; 2 Department of Microbiology, College of Basic Medical Sciences, Dalian Medical University, Dalian, China; 3 Puensum Genetech Institute, Wuhan, China; 4 School of Traditional Chinese Medicine, Beijing University of Chinese Medicine, Beijing, China; 5 Department of Traditional Chinese Medicine, Beijing Friendship Hospital, Capital Medical University, Beijing, China; 6 Department of Acupuncture and Moxibustion, Beijing Hospital of Traditional Chinese Medicine, Capital Medical University, Beijing, China; 7 State Key Laboratory of Dampness Syndrome of Chinese Medicine, The Second Affiliated Hospital of Guangzhou University of Chinese Medicine, Guangzhou, China; 8 Key Laboratory of Health Cultivation of the Ministry of Education, Beijing University of Chinese Medicine, Beijing, China

**Keywords:** Osteoarthritis, Gouty arthritis, Gut virome, Virus-like particle-based metagenome, Viral diversity, Viral dysbiosis

## Abstract

**Background/purpose(s):**

The gut microbiota and its metabolites play crucial roles in pathogenesis of arthritis, highlighting gut microbiota as a promising avenue for modulating autoimmunity. However, the characterization of the gut virome in arthritis patients, including osteoarthritis (OA) and gouty arthritis (GA), requires further investigation.

**Methods:**

We employed virus-like particle (VLP)-based metagenomic sequencing to analyze gut viral community in 20 OA patients, 26 GA patients, and 31 healthy controls, encompassing a total of 77 fecal samples.

**Results:**

Our analysis generated 6819 vOTUs, with a considerable proportion of viral genomes differing from existing catalogs. The gut virome in OA and GA patients differed significantly from healthy controls, showing variations in diversity and viral family abundances. We identified 157 OA-associated and 94 GA-associated vOTUs, achieving high accuracy in patient-control discrimination with random forest models. OA-associated viruses were predicted to infect pro-inflammatory bacteria or bacteria associated with immunoglobulin A production, while GA-associated viruses were linked to Bacteroidaceae or Lachnospiraceae phages. Furthermore, several viral functional orthologs displayed significant differences in frequency between OA-enriched and GA-enriched vOTUs, suggesting potential functional roles of these viruses. Additionally, we trained classification models based on gut viral signatures to effectively discriminate OA or GA patients from healthy controls, yielding AUC values up to 0.97, indicating the clinical utility of the gut virome in diagnosing OA or GA.

**Conclusion:**

Our study highlights distinctive alterations in viral diversity and taxonomy within gut virome of OA and GA patients, offering insights into arthritis etiology and potential treatment and prevention strategies.

**Graphical Abstract:**

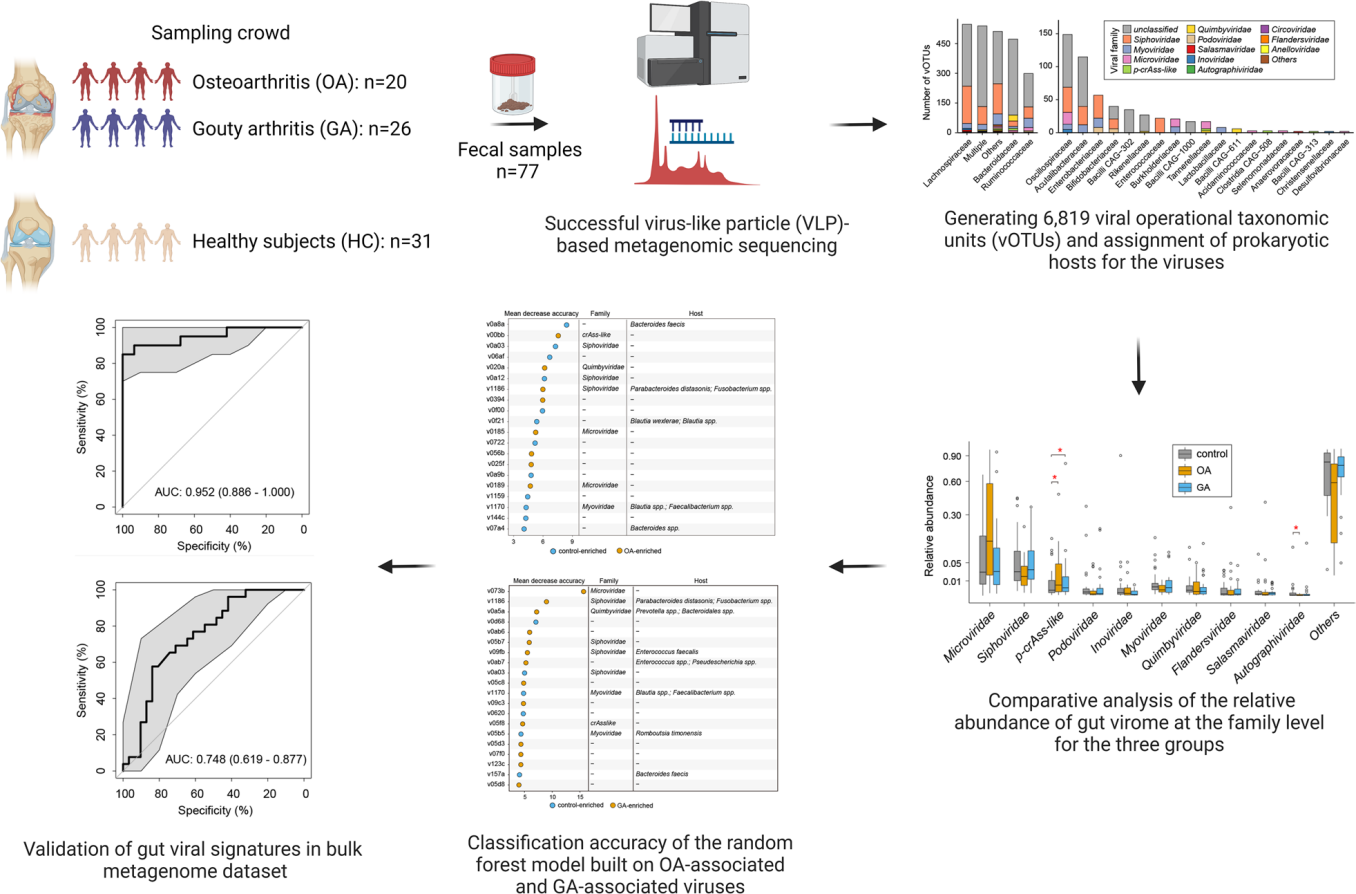

**Supplementary Information:**

The online version contains supplementary material available at 10.1186/s12967-024-05374-6.

## Introduction

Arthritis is a globally prevalent condition characterized by osteochondral destruction, synovial swelling, inflammation, and joint stiffness [1]. Osteoarthritis (OA) and gouty arthritis (GA) are two common forms of arthritis, yet their exact etiology remains unknown, and no cure currently exists [2, 3]. Current treatments, primarily anti-inflammatory drugs, come with significant side effects like gastric bleeding and cardiovascular risks, underscoring the need for deeper pathogenic understanding and innovative therapeutic strategies. Notably, OA, the most common arthritis type, is a significant pain and disability source in the elderly [4]. Recent studies have demonstrated that gut microbes can influence bone stability and accelerate OA's onset and progression [5]. GA, distinguished by its recurrent inflammation, hyperuricemia, and urate crystal accumulation, also shows connections to gut microbiota changes [6]. Growing evidence suggests that gut microbiota influences purine metabolism, urate excretion, and NLRP3 inflammasome activation, thereby contributing to GA pathogenesis [6]. These findings suggest a potential association between gut microbiota and arthritis, suggesting that microbiota might be a disease trigger.

Human gut microbiota comprising bacteria, fungi, viruses, and other entities, playing roles in nutrient metabolism, immune responses, and pathogen defense [7]. Gut viruses are increasingly recognized for their impact on bacterial diversity and horizontal gene transfer [8]. Recent studies have highlighted the association between gut viruses and several autoimmune and metabolic diseases, such as rheumatoid arthritis (RA), systemic lupus erythematosus (SLE), ankylosing spondylitis (AS), Inflammatory bowel disease (IBD), metabolic syndrome (MetS), and OA [9–14]. In patients with IBD, gut virome dysbiosis, mainly attributed to the proliferation of Caudovirales, has been demonstrated to affect intestinal immunity and barrier functions, thus influencing overall intestinal homeostasis [15]. Besides, the discovery of the temperate Ca. Heliusviridae serves as a foundation for investigations into the impact of phages on gut bacteria and their role in patients with MetS [13]. Additionally, perturbations in viral composition and microbial correlation network in oral and gut samples of RA patients may be linked to RA pathogenicity [16]. Specifically, in patients with RA, high-risk individuals harbor unique sets of auxiliary metabolic genes carried by gut bacteriophages, which correlate with anti-CCP status. This implies a direct impact of these bacteriophages on the metabolic and immunomodulatory functions of the gut microbiota [17]. Therefore, investigating changes in gut virome diversity and structure is crucial for understanding the underlying pathogenesis of diseases.

Our study aimed to characterize the gut virome in OA, GA patients, and healthy controls. By enhancing our understanding and distinguishing the etiology and pathogenesis of different forms of arthritis based on gut virome exploration, we anticipate the development of novel prevention and treatment strategies for arthritis.

## Materials and methods

### Participants and sample collection

The research protocol was approved by the Medical Ethical Committees of the Second Affiliated Hospital of Guizhou University of Traditional Chinese Medicine (approval numbers KY2023001 and KYW2023005). All subjects who participated in this research provided written informed consent. This study enrolled 20 OA and 26 GA patients admitted to the Department of Rheumatology and Immunology at the Second Affiliated Hospital of Guizhou University of Chinese Medicine, China, from August 2020 to August 2021. All patients with OA met the criteria outlined in the 2019 edition of the Chinese guidelines for diagnosing and treating osteoarthritis, while all patients with GA fulfilled the guidelines of 2015 American College of Rheumatology/European League Against Rheumatism (ACR/EULAR) classification criteria [18]. Thirty-one healthy controls were recruited from the same hospital based on the previously described methods [19] (Table 1). All participants self-collected their fecal samples post-defecation at the hospital, which were then promptly placed on dry ice. The samples were taken to the lab, separated into two portions, and stored in a pair of freezing tubes. These fecal specimens were then preserved at − 80 °C.

**Table 1:** Phenotypic and clinical characteristics of subjects recruited in this study

Parameters	OA patients (n = 20)	GA patients (n = 26)	*p*-value (OA vs. GA)	HC (n = 31)	*p*-value (OA vs. HC)	*p*-value (GA vs. HC)
Gender, F/M	16/4	1/25	< 0.001	12/19	0.0047	0.0032
Age, years	51.8 ± 12.8	45.2 ± 8.8	0.059	47.5	0.226	0.399
BMI, kg/m^2^	21.9 ± 3.1	22.7 ± 3.3	0.254	23.2 ± 2.8	0.178	0.610
ESR, mm/h	14.5 ± 10.4	34.7 ± 27.5	0.0022			
CRP, mg/dl	2.0 ± 3.0	10.4 ± 11.1	0.0013			
DAS28 score	4.3 ± 1.4	4.6 ± 1.1	0.504			
RF, IU/ml	22.0 ± 33.7	13.6 ± 9.5	0.349			
Anti-CCP, U/ml	27.1 ± 40.9	15.9 ± 31.6	0.463			
IgM, RU/ml	12.2 ± 15.0	9.4 ± 8.2	0.548			
IgG, RU/ml	4.7 ± 5.4	5.2 ± 5.4	0.834			
IgA, RU/ml	7.6 ± 10.4	5.8 ± 3.9	0.546			
PLT, × 10^9^/l	195 ± 66	273 ± 108	0.0055			
Disease duration, months	79 ± 81	135 ± 68	0.020			

The data are presented as the mean ± SD

*HC* healthy controls, *BMI* body mass index, *ESR* erythrocyte sedimentation rate, *CRP* C-reactive protein, *RF* rheumatoid factor, *Anti-CCP* anticyclic citrullinated peptide antibody, *IgM* immunoglobulin M, *IgG* immunoglobulin G, *IgA* immunoglobulin A, *PLT* blood platelet count

### Virus-like particle (VLP) enrichment, metagenomic DNA extraction and sequencing

VLP enrichment was carried out following our established protocols with slight modifications [20]. Briefly, 0.17 g of fecal material from each subject was mixed with 1 ml of Hank’s Balanced Salt Solution (HBSS) and thoroughly homogenized. The homogenized samples were centrifuged (10,000*g*, 10 min, 4 ℃) and the resulting supernatant was sequentially filtered through 0.45 μm and 0.2 μm filters. Subsequently, ultracentrifugation (750,000*g*, 1 h, 8 ℃) was performed and the resulting pellet was suspended in 500 μL of HBSS. A portion (120 μL) of this suspension was subjected to nuclease treatment, comprising 2.4 μL of TURBO DNase (4.8 U, Invitrogen), 8 μL of RNase A/T1 Mix (16 μg RNase A, 40 U RNase T1, Thermo Scientific), and 1 μL of Benzonase (5 U, EMD Millipore), for 120 min at 37 ℃. Subsequent nucleic acid extraction was performed using the TIANamp Viral Genome DNA/RNA extraction kit (TIANGEN, China) according to the manufacturer's instructions. The DNA sequencing library was prepared using the NEB Next Ultra DNA Library Prep Kit (NEB, USA) as per the manufacturer's protocol, with unique index codes assigned to each sample. Library quality was assessed using an Agilent 2100 instrument. Index-coded samples were clustered using the Illumina PE Cluster Kit (Illumina, USA) on a cBot Cluster Generation System following manufacturer's instructions. Following cluster generation, metagenome shotgun sequencing was conducted on the Illumina NovaSeq platform, yielding 150 bp paired-end reads.

For bulk metagenomic sequencing, microbial DNA was extracted from each fecal specimen (~ 0.17 g) using the TIANamp Stool DNA Kit (TIANGEN, China) according to the manufacturer's instructions. DNA quality was evaluated using the Nanodrop and Qubit 2.0 system.

### Bioinformatic analysis

The clean reads from each sample underwent assembly into contigs using MEGAHIT with the following parameters: “-k-list 21, 41, 61, 81, 101, 121, 141” [21]. Contigs with a length of ≥ 5 kb were selected for the identification of viral sequences. Identification, decontamination, and dereplication of viral sequences were conducted following established protocols [12, 22, 23]. In brief, the assembled contigs underwent a stringent quality assessment: (1) contigs were classified as viral if their viral gene content exceeded that of microbial genes, as determined by CheckV [24], (2) contigs showing a *p*-value of less than 0.01 and a score exceeding 0.90 in DeepVirFinder [25] were considered viral, or (3) viral sequences were identified using default settings in VIBRANT [26]. To ensure the purity of the viral sequences, a decontamination step was implemented based on the identification of bacterial universal single-copy orthologs (BUSCO) within each viral sequence, following a previously described approach [27]. Sequences with a BUSCO ratio greater than 5% were eliminated. Pairwise comparison of all viral sequences was conducted using BLASTN [28], and viruses with a shared nucleotide identity of 95% or greater across at least 75% of their sequences were clustered together to form a viral operational taxonomic unit (vOTU). The quality of viral sequences was assessed using CheckV [24]. Taxonomic annotation of viral sequences was performed through protein sequence alignment against a comprehensive database, which included resources from the Virus-Host DB [29] and viral proteins from crAss-like phage, Flandersviridae, Quimbyviridae, and Gratiaviridae. A viral sequence was assigned a family-level classification when over a quarter of its proteins showed a match to the same viral family. Virus-host predictions were carried out using two bioinformatic methodologies: CRISPR-spacer matches and prophage blasts, following established methodologies [20]. Functional annotation of viral proteins was performed based on the Kyoto Encyclopedia of Genes and Genomes (KEGG) database [30], with each protein receiving a KO assignment based on the best-hit gene in the database.

### Statistical analysis

Statistical analysis and visualization were conducted under the R platform. Gut virome diversities were estimated at the vOTU level. The observed number of vOTUs was calculated as the count of vOTUs with a relative abundance greater than 0. The Shannon and Simpson diversity indexes were computed using the diversity function from the *vegan* package [31]. Bray–Curtis distances between samples were calculated with the *vegdist* function. Principal coordinates analysis (PCoA) of Bray–Curtis distances was performed using the *pcoa* function from the *vegan* package. Permutational multivariate analysis of variance (PERMANOVA) was conducted using the *adonis* function. Viral markers at the vOTU level were identified between patients and healthy pregnant women using the *wilcox.test* function from the R *stats* package. The resulting *P* values were adjusted using the *p.adjust* function with the "method = BH" option to generate the *q*-value. Random forest models based on viral or bacterial markers were constructed using the *randomForest* package, followed by fivefold cross-validation. Model performance was evaluated based on the area under the curve (AUC), calculated using the *roc* function. The importance ranking of markers was determined using the *importance* function.

## Results

### Subjects, sequencing, and viral identification

**Fig. 1:**
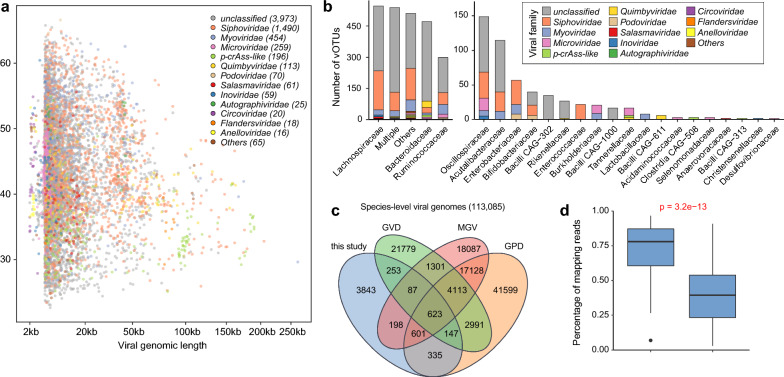
Characteristics of gut viral genomes used in this study. **a** Distribution of viral genome size and GC content. The point color represents viral family-level annotation. **b** Assignment of prokaryotic hosts for the viruses. **c** Overlap of species-level viral genomes among the four gut viral databases. **d** Performance comparison in terms of the read recruitment between our viral catalog and the combined public catalog. Statistical significance was determined using the Wilcoxon rank-sum test

All 77 fecal samples were performed VLP-based metagenomic sequencing and obtained 348.4 Gbp (4.5 ± 1.4 Gbp per sample; Table S1) high-quality non-human data. The reads were de novo assembled into 140,623 contigs with a minimum length of 2000 bp, of which 11,800 contigs were recognized as credible viral sequences using an integrated homology- and feature-based pipeline (see Materials and methods). These virus sequences were clustered at the species level (> 95% nucleotide similarity and > 75% coverage) to generate 6819 viral operational taxonomic units (vOTUs) (Fig. 1a). The average length of vOTUs was 16,026 bp, with an N50 length of 28,443 bp and the maximum length of 262,173 bp (Fig. S1a). The completeness of the vOTUs using CheckV were evaluated [24], revealing 12.2% complete, 6.3% high-, and 9.1% medium-, and 72.4% low-quality or not-determined viruses (Fig. S1b).

Taxonomically, 41.74% vOTUs were dominated by Siphoviridae, Myoviridae, and Microviridae (Fig. 1a)*.* Most of the remaining family-level annotated vOTUs belonged to members of the crAss-like phages, Quimbyviridae, Podoviridae, and so on. In addition, we performed host prediction of the vOTUs based on their homology of genome sequences or CRISPR spacers to species-level prokaryotic genomes from the Unified Human Gastrointestinal Genome (UHGG) collection, and predicted the hosts of 45.5% of vOTUs. Consistent with previous findings [23, 27], the majority of vOTUs were predicted to infect Firmicutes and Bacteroidia (Fig. 1b). At the family level, vOTUs were dominated by Lachnospiraceae, Bacteroidaceae and Ruminococcaceae.

To assess how representative our vOTUs catalog is of the gut viral composition, we compared the vOTUs with several existing large-scale human gut virus catalogs, including the Gut Virome Database (GVD) [27], Gut Phage Database (GPD) [8], and Metagenomic Gut Virus (MGV) [32]. There were 56.4% of vOTUs did not overlap with the viral genomes from other existing catalogs (Fig. 1c), indicating a substantial novelty of viral genomes in our catalog. Furthermore, the high-quality sequencing data were mapped against 6,819 vOTUs, leading to an average read mapping rate of 72.5% (Fig. 1d). Notably, this rate was only 39.4% when the sequencing data were mapped against combination of three existing gut virus catalogs. These findings suggested that the viral genomes in our catalog substantially improved viral read recruitment in understudied VLP-based virome samples.

### Gut virome diversity and structure associated with arthritis

**Fig. 2:**
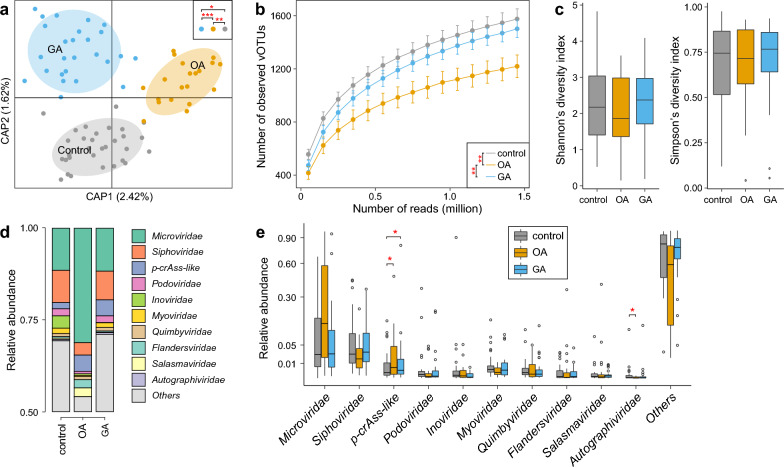
Comparison of the gut virome diversity and structure among healthy controls, patients with osteoarthritis, and patients with gouty arthritis.** a** Principal coordinates analysis (PCoA) based on Bary-Curtis distance of the vOTU-level profiles. Statistical significance, estimated by permutational multivariate analysis of variance (PERMANOVA), was indicated in the upper right corner. *, *adonis p* < 0.05; **, *adonis p* < 0.01; ***, *adonis p* < 0.001. **b** The rarefaction curve of the observed vOTUs as the number of mapping reads increases. Statistical significance was determined using the Wilcoxon rank-sum test. **p < 0.01. **c** Comparison of alpha diversity indexes of the gut virome among the three groups. **d** Gut virome composition at the family level for the three groups. **e** Comparative analysis of the relative abundance of viral families. Statistical significance was determined using the Wilcoxon rank-sum test. *p < 0.05

To illustrate the alteration of gut virome in OA or GA patients, we compared the viral community diversity and compositional structure among three groups. PCoA plot showed slightly separation between OA patients and healthy controls (Fig. 2a). Furthermore, PERMANOVA analysis confirmed that the overall viral community in healthy controls at the vOTU level was significantly different with that in both OA (*adonis* R^2^ = 1.2%, *p* = 0.002) and GA patients (*adonis* R^2^ = 0.5%, *p* = 0.031). Notably, the viral community difference between OA and GA patients (*adonis* R^2^ = 1.7%, *p* < 0.001) was relatively larger than the differences between healthy controls and two disease groups, suggesting distinct characteristics of the gut viral composition in OA and GA patients.

For alpha diversity, OA patients showed a significant decrease in gut viral richness (i.e., the number of observed vOTUs) than healthy controls and GA patients (Wilcoxon rank-sum test *p* < 0.01), while there was no statistical difference in viral richness between healthy controls and GA patients (Fig. 2b). Besides, no significant difference was observed among three groups in the gut viral diversity indexes including Shannon and Simpson indexes (Fig. 2c).

We next investigated the alterations of gut viral composition at family level among arthritis patients and healthy controls. Visible differences were observed for the relative abundances of several dominant viral families among three groups (Fig. 2d). The family crAss-like phages had a significantly higher abundance in both OA and GA patients than in healthy controls (Wilcoxon rank-sum test* p* < 0.05), while Autographiviridae showed a reduction in OA patients compared with healthy controls (*p* = 0.015; Fig. 2d, e; Table S2). Also, the family Microviridae displayed an approached significance increase in OA patients compared with GA patients and healthy controls (Wilcoxon rank-sum test* p* = 0.078 and 0.068, respectively), while Siphoviridae were less abundant in OA patients than healthy controls (*p* = 0.052). The relative abundances of 4 viral families, including Podoviridae, Inoviridae, Myoviridae, and Quimbyviridae, were slightly reduced in OA patients compared with those in healthy controls (Table S2)*.* These findings suggest potential interactions between these viral families and arthritis diseases.

### Gut viral signatures of the OA patients

**Fig. 3:**
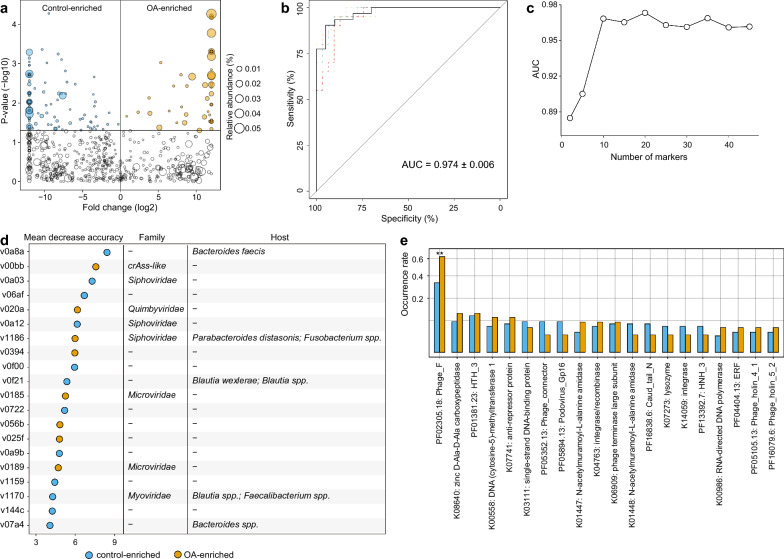
Identification and functional characteristics of gut viral signatures associated with osteoarthritis (OA).** a** Volcano plot displaying the statistical differences in the relative abundances of vOTUs between OA patients and healthy controls. The x-axis represented the log2-transformed fold changes, while the y-axis represented the log10-transformed p-values obtained from the Wilcoxon rank-sum test. The yellow points indicated vOTUs enriched in OA patients, while the blue points indicated vOTUs enriched in healthy controls. **b** Classification accuracy of the random forest model built on all OA-associated viruses in distinguishing patients from healthy controls. The classification accuracy was quantified as the area under the receiver operating characteristic curve (AUC) and validated using 5 × fivefold cross-validation. **c** Classification accuracy of the random forest model built on different amounts of OA-associated viruses in distinguishing patients from healthy controls. **d** The top 20 OA-associated viruses with the highest mean decrease accuracy were obtained from the random forest model. **e** Comparative analysis of the occurrence rates of functional orthologs between OA- and control-enriched vOTUs. Functional orthologs starting with the letter “K” represented KEGG functional orthologs, while those starting with the letter “P” represented Pfam families. Statistical significance was determined using Fisher's exact test. **p < 0.01

To investigate the OA-associated gut viral signatures, we conducted a comparison of the viral compositions at the vOTU level between OA patients and healthy controls. In total, 157 vOTUs were identified with statistical differences in relative abundances between two groups (Wilcoxon rank-sum test *p* < 0.05, corresponding to *q* < 0.122; Fig. 3a; Table S3), in the case of 681 core vOTUs with relatively high abundance (average relative abundance > 0.1%, which represented 92.3% of total sequences in all analyzed samples). Among these differential viruses, 48 vOTUs were abundant in the OA patients, while 109 were enriched in healthy controls. 19 of 48 OA-enriched were members of Microviridae, consistent with the enrichment of Microviridae in the family-level results. Besides, a large proportion of OA-enriched vOTUs were predicted to infect Bacteroidaceae or Tannerellaceae, while the Lachnospiraceae and Ruminococcaceae phages were frequent in the control-enriched vOTUs compared with OA-enriched vOTUs (Fig. S2).

We performed the importance assessment of these viral signatures using a random forest model with 5 × fivefold cross-validation, which showed an outstanding potential for the classifiability of patients versus healthy controls (area under the receiver operating characteristic curve [AUC] = 0.974; Fig. 3b). Strikingly, a new model trained using the most important 20 vOTUs achieved a cross-validation AUC > 0.97 (Fig. 3c); these vOTUs are the most representative signatures of OA virome. Twelve of representative vOTUs were enriched in healthy controls, including two viruses, v0a8a and v07a4, that were predicted to infect the members of *Bacteroides*, particularly *B. faecis* that had been linked to Immunoglobulin A (IgA) production in human gut [33]. Two viruses, v0f21 and v1170, were predicted to infect some butyrate-producing bacteria with anti-inflammatory properties, including the members of *Blautia* and *Faecalibacterium* [34]. By contrast, 8 of the representative vOTUs were enriched in OA patients, including 2 Microviridae, 1 crAss-like phages, and 1 Quimbyviridae viruses. Notably, a Microviridae member v0185 carried a gene encoding the *Chlamydia* phage Chp2 scaffold protein (Fig. S3), suggesting its possibility of infecting *Chlamydia* species that were commonly related to reactive arthritis [35]. This observation is consistent with previous findings that have shown an increased presence of crAss-like phages in the gut virome of patients with RA and other autoimmune diseases [9]. Besides, an OA-enriched viruses v1186 tended to infect the harmful *Fusobacterium* spp. that may induce inflammation and suppress host immunity [36]. Collectively, these findings suggested that OA-associated viruses may link to the mechanisms or functions of OA, possibly via their prokaryotic hosts.

To elucidate the functional characteristics of the gut viral signatures, we searched the predicted proteins from 157 OA-associated vOTUs against KEGG [30] and Pfam protein families database [37], generating 320 annotated protein families (Table S4). Most annotated proteins were assigned into typical viral functions involving phage structural components, genetic information processing, and peptidoglycan metabolism. We compared the occurrence rate of functional orthologs between the OA-enriched or control-enriched vOTUs. Among the 20 most common functional orthologs, only a phage capsid protein (PF02305.18) exhibited a higher frequency in OA-enriched vOTUs when compared with healthy controls (Fig. 3e). Also, no statistically significant differences were observed between the OA-enriched and HC-enriched vOTUs in terms of the occurrence rates of auxiliary metabolic genes (AMGs) (Table S4).

### Gut viral signatures of the GA patients

**Fig. 4:**
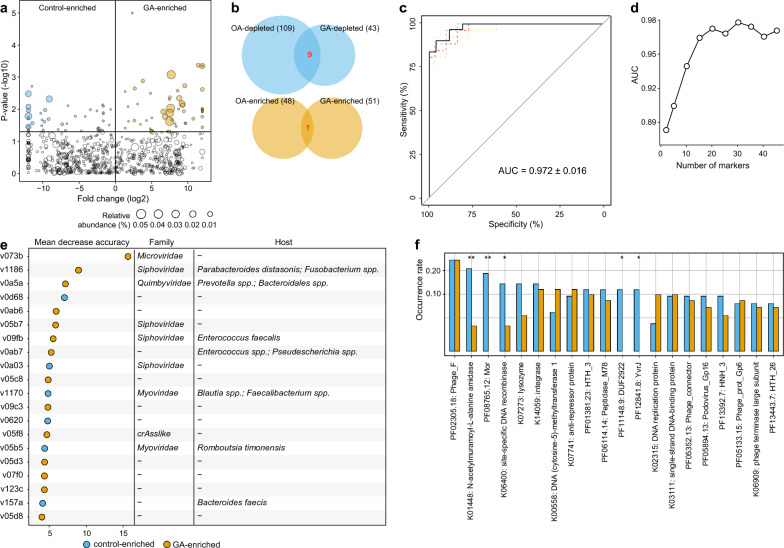
Identification and functional characteristics of gut viral signatures associated with gouty arthritis (GA).** a** Volcano plot displaying the statistical differences in the relative abundances of vOTUs between GA patients and healthy controls. The x-axis represented the log2-transformed fold changes, while the y-axis represented the log10-transformed p-values obtained from the Wilcoxon rank-sum test. The yellow points indicated vOTUs enriched in GA patients, while the blue points indicated vOTUs enriched in healthy controls. **b** Overlap of GA- and OA-associated vOTUs. **c** Classification accuracy of the random forest model built on all GA-associated viruses in distinguishing patients from healthy controls. The classification accuracy was quantified as the area under the receiver operating characteristic curve (AUC), and validated using 5 × fivefold cross-validation. **d** Classification accuracy of the random forest model built on different amounts of GA-associated viruses in distinguishing patients from healthy controls. **e** The top 20 GA-associated viruses with the highest mean decrease accuracy were obtained from the random forest model. **f** Comparative analysis of the occurrence rates of functional orthologs between GA- and control-enriched vOTUs. Functional orthologs starting with the letter “K” represented KEGG functional orthologs, while those starting with the letter “P” represented Pfam families. Statistical significance was determined using Fisher's exact test. *p < 0.05, **p < 0.01

Next, we compared the gut viral compositions at the vOTU level between 26 GA patients and 31 healthy controls. Among 745 core vOTUs with relatively high abundance (average relative abundance > 0.1%, which represented 91.7% of total abundance in all analyzed samples), 94 vOTUs significantly differed in relative abundances between two groups (Wilcoxon rank-sum test *q* < 0.05; Fig. 4a; Table S5). 51 of these differential vOTUs were more abundant in the GA patients, while 43 were enriched in healthy controls. The GA-enriched vOTUs included 8 members of Siphoviridae, 7 Microviridae, 2 Quimbyviridae, and 1 crAss-like phages, while the control-enriched vOTUs included 8 Siphoviridae, 3 Microviridae, 2 Myoviridae. Similar to OA-associated viral signatures, Bacteroidaceae phages were more frequent in the GA-enriched vOTUs compared with control-enriched vOTUs, while Lachnospiraceae phages were more frequent in control-enriched vOTUs (Fig. S4). Noticeably, we found the viral signatures of OA and GA are markedly separated. Only one vOTU (v1186, predicted to infect *Parabacteroides distasonis* and *Fusobacterium*) was shared between the OA-enriched and GA-enriched vOTUs, and only 9 vOTUs were enriched in healthy controls versus OA or GA patients (Fig. 4b). These findings agreed with the abovementioned results suggesting distinct characteristics of the gut virome in OA and GA patients.

Random forest model based on the GA-associated vOTUs achieved an AUC of 0.972 for classifying the GA patients from healthy controls (Fig. 4c). Also, the model obtained high performance (AUC > 0.97) when using the most important 20 vOTUs as the representative signatures of GA virome (Fig. 4d). 14 of the 20 representation vOTUs were enriched in GA patients, which included a Quimbyviridae that predicted to infect *Prevotella* and *Bacteroidales* spp. and 2 vOTUs (v09fb and v0ab7) that can infect the *Enterococcus* species (Fig. 4e). The control-enriched vOTUs contained v1170 (which is predicted to infect some butyrate-producing bacteria), a *Romboutsia timonensis* phage v05b5 and a *Bacteroides faecis* phage v157a.

Functionally, we annotated 366 protein families from 94 GA-associated vOTUs and compared the occurrence rate of these functions between the GA-enriched and control-enriched vOTUs (Table S6). Five of the 20 most common functional orthologs, including K01448 (an N-acetylmuramoyl-L-alanine amidase), PF08765.12 (a middle operon regulator), K06400 (a site-specific DNA recombinase), PF11148.9, and PF12841.8, were significantly differed and all were rare occurred in the GA-enriched vOTUs (Fig. 4f). Besides, the GA-enriched Quimbyviridae vOTU *v0a5a* carried a gene encoding the hemolysin-related protein (Fig. S5).

### Validation of gut viral signatures based on bulk metagenome dataset

**Fig. 5:**
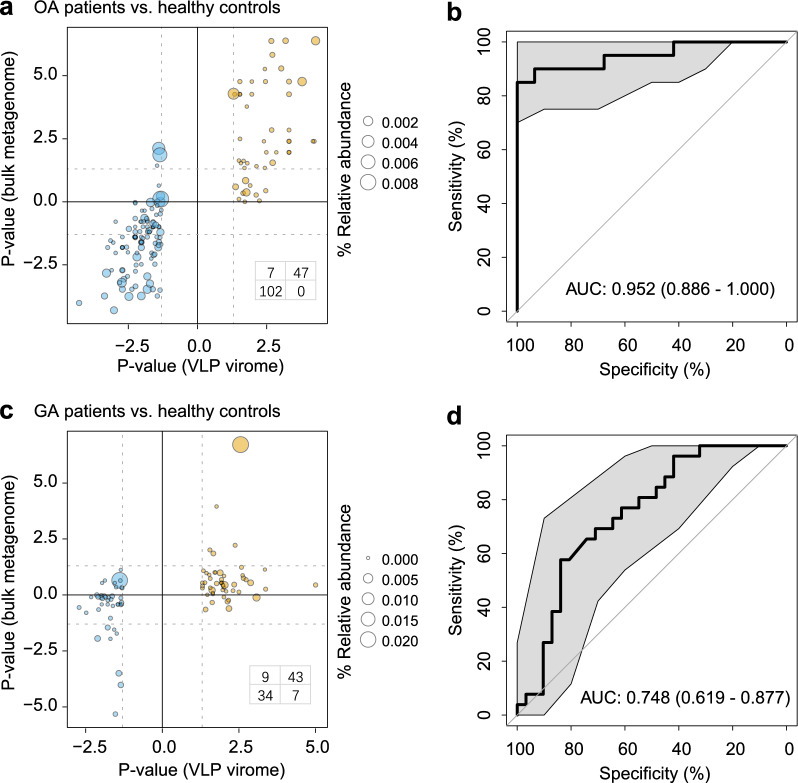
Validation of gut viral signatures in bulk metagenome dataset. **a** Statistical significance of 157 OA-associated vOTUs in VLP and bulk metagenome dataset, respectively. The plot displayed yellow points for vOTUs enriched in patients and blue points for vOTUs enriched in healthy controls. The x-axis represented the log10-transformed p-values obtained from the Wilcoxon rank-sum test, while the y-axis represented the log10-transformed p-values. The transformed p-value was further converted to a negative value if a vOTU showed a higher relative abundance in healthy controls compared to patients in the corresponding metagenome dataset. The table in the lower right corner indicated the number of vOTUs in each quadrant. **b** Classification accuracy of the random forest model built on 157 OA-associated viruses in distinguishing OA patients from healthy controls in the bulk metagenome dataset. The classification accuracy was quantified as the area under the receiver operating characteristic curve (AUC). **c** The statistical significances of 94 GA-associated vOTUs in VLP and bulk metagenome dataset, respectively. **d** Classification accuracy of the random forest model built on 94 GA-associated viruses in distinguishing GA patients from healthy controls in the bulk metagenome dataset

To test whether the gut virome signatures of arthritis characterized from the VLP-based virome dataset are also present to at least some extent in the whole metagenome dataset, we analyzed the bulk metagenomes from fecal samples of all 77 subjects, representing a total of 724.4 Gbp (9.4 ± 2.3 Gbp per sample; Table S1) data for analyses. Compared with VLP-based virome’s results, 94.9% (149/157) of OA-associated vOTUs exhibited consistent enrichment or depletion in patients and healthy controls in the bulk metagenome dataset (Fig. 5a). In particular, 38 and 63 of these vOTUs were significantly enriched in the OA patients and healthy controls, respectively, in bulk metagenomes with consistent trends in VLP-based viromes. Likewise, a random forest classifier trained by the relative abundances of OA-associated vOTUs from the VLP-based virome dataset generated an AUC of 0.952 in discriminating OA patients from healthy controls under the bulk dataset (Fig. 5b). Similar to GA, 83.0% (78/94) of the GA-associated vOTUs were reproducible, at least in terms of the trend, in bulk metagenomes compared with the results from VLP-based viromes (Fig. 5c). And a random forest classifier trained by the GA-associated vOTUs from the VLP-based virome dataset generated an AUC of 0.748 in discriminating patients from healthy controls under the bulk dataset (Fig. 5d). These findings highly indicate repeatability and predictive power of the gut viral signatures of OA and GA under different technologies.

### Medications have a limited impact on the gut virome in patients

Medications have been widely reported to have considerable influences on gut microbiota [38, 39]. PERMANOVA analysis of the gut viral profiles of OA and GA patients were performed to investigate the impact of medications on the arthritis gut virome. For OA patients, we found that the usage of Meloxicam has a significant impact on the gut virome (*adonis p* = 0.012; Table S7). However, this effect was relatively smaller than the effect of disease-healthy stratification (Fig. 1a); and also, the usage of drugs had no significant impact on the gut virome (*adonis p* = 0.530). For GA patients, none of the four medications, including Colchicine, Febuxostat, Etoricoxib, and Methylprednisolone, had a significant impact on the gut virome (*adonis p* > 0.05; Table S7).

## Discussion

Our study fills a research gap by examining the role of gut viruses in arthritis, specifically in OA and GA patients. Using VLP-based metagenomic sequencing, we enhanced the detection of previously missed viral diversity and species, and shed light on the interplay between gut viruses and bacteria. A notable finding was the increased proportion of Microviridae, which predominantly target Gram-negative bacteria [40], and has been implicated in various conditions, including IBD, irritable bowel syndrome (IBS), and coronary heart disease [41–44]. Our results suggested an elevated prevalence of Microviridae in arthritis patients, deviating from typical stool virome. Consistent with previous studies, our data also revealed that the majority of these vOTUs are likely to infect bacterial families crucial for gut health, specifically Lachnospiraceae, which are important in carbohydrate metabolism and are linked to autoimmune diseases [17, 45].

Our analysis also unveiled distinct gut viromes in OA and GA patients, notably a higher abundance of crAss-like phages compared to healthy controls. CrAss-like phages are a newly discovered clade of viruses commonly found in the human gut. They can infect bacteria of the phylum Bacteroidetes and potentially play a beneficial role in the gut ecosystem [46, 47]. However, the enrichment of crAss-like phages has also been observed in the gut viromes of other autoimmune diseases, such as SLE [12]. Our findings, along with these results, suggest a unique role for these phages in immune-related diseases that merits further research.

We identified 157 vOTUs with differential abundances between OA patients and healthy controls. Several OA-depleted vOTUs that were predicted to infect *B. faecis* (involving immunoglobulin A production in human gut [33]) and anti-inflammatory butyrate-producing bacteria *Blautia* and *Faecalibacterium* spp. [48, 49], while OA-enriched vOTUs were associated with *Fusobacterium* species. For GA, vOTUs targeted *Fusobacterium*, *Prevotella*, and *Enterococcus*. were identified. Fusobacterium is an important pro-inflammatory bacterium in the human gut that may induce inflammation and suppress host immunity [36] and contribute to the development of colorectal adenomas and carcinomas [36, 50]. It is also significantly enriched in the gut of patients with other systemic diseases such as chronic kidney disease [51] and ulcerative colitis [52]. Therefore, the enrichment of Fusobacterium phages in the gut of OA and GA patients may indicate harmful effects that need further validation. Furthermore, comparative analyses of viral functions identified numerous significantly different functional orthologs. For instance, a GA-enriched Quimbyviridae vOTU carried a gene encoding the hemolysin-related protein, which may cause rapid chondrocyte death [53]. These findings suggested potential mechanistic links between disease-associated vOTUs and the disease itself, although the exact roles of these viruses remain to be elucidated.

Furthermore, the influence of medications on the OA and GA viromes was examined, as medications have been reported to influence the gut microbiota. Notably, Meloxicam, an NSAID utilized for OA management [54], demonstrated a modest yet significant effect on the gut virome of OA patients, diverging from other examined drugs. Meloxicam may affect the homeostasis and function of the gut virome through multiple mechanisms, including immunomodulation, regulation of the gut microbiota, direct impact on viral lifecycle, and alteration of the gut environment. Additionally, NSAIDs may indirectly impact the gut ecosystem by altering environmental factors such as intestinal pH, mucosal layer, and nutrient utilization [55]. In GA patients, none of the medications had a significant impact on the gut virome, allowing us to focus on the differences in the gut virome specifically associated with the disease itself.

One notable limitation of the present study is the relatively small sample size and the viral databases used are not exhaustive. For a thorough understanding of the gut virome's link to arthritis, larger patients and control cohorts are essential.

In conclusion, we revealed and distinguished the alterations in viral diversity and taxonomic composition in the gut virome of patients with OA and GA. Further research on the etiology of arthritis and the gut viral community will provide new perspectives for the treatment and prevention of arthritis.

### Supplementary Information

Supplementary Material 1.

Supplementary Material 2.

Supplementary Material 3.

Supplementary Material 4.

Supplementary Material 5.

Supplementary Material 6.

## Data Availability

The raw metagenomic sequencing dataset for this study has been deposited in the European Nucleotide Archive (ENA) at EMBL-EBI under accession number PRJEB52499 (https://www.ebi.ac.uk/ena/data/view/PRJEB52499) and PRJEB58696 (https://www.ebi.ac.uk/ena/data/view/PRJEB58696). The authors declare that all other data supporting the findings of the study are available in the main text and supplementary materials, or from the corresponding author upon request.
