# Supplementary figures and images for: Distinct characteristics of the gut virome in patients with osteoarthritis and gouty arthritis
Source: J Transl Med. 2024 Jun 13;22:564. doi: 10.1186/s12967-024-05374-6 (PMC11170907; doi:10.1186/s12967-024-05374-6)

**a**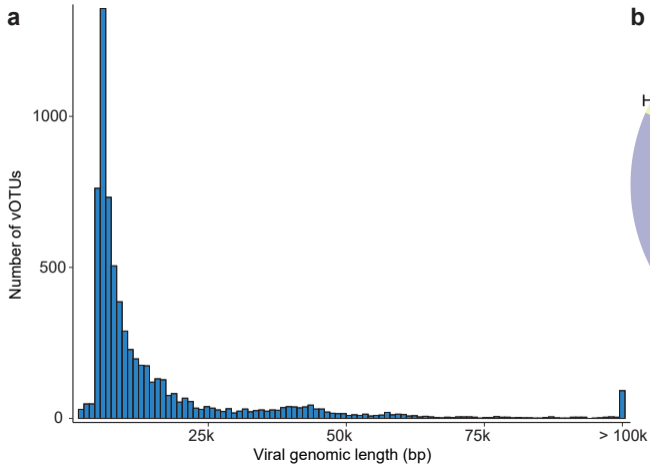**b**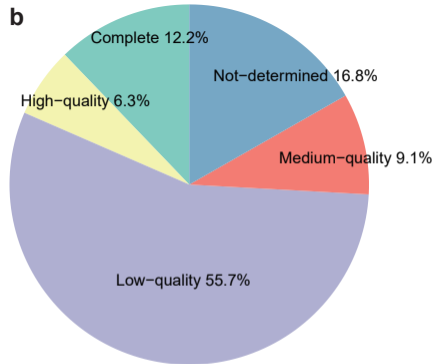

Supplement: Supplementary file 1 — Supplementary Material 1. [file 12967_2024_5374_MOESM1_ESM.pdf]

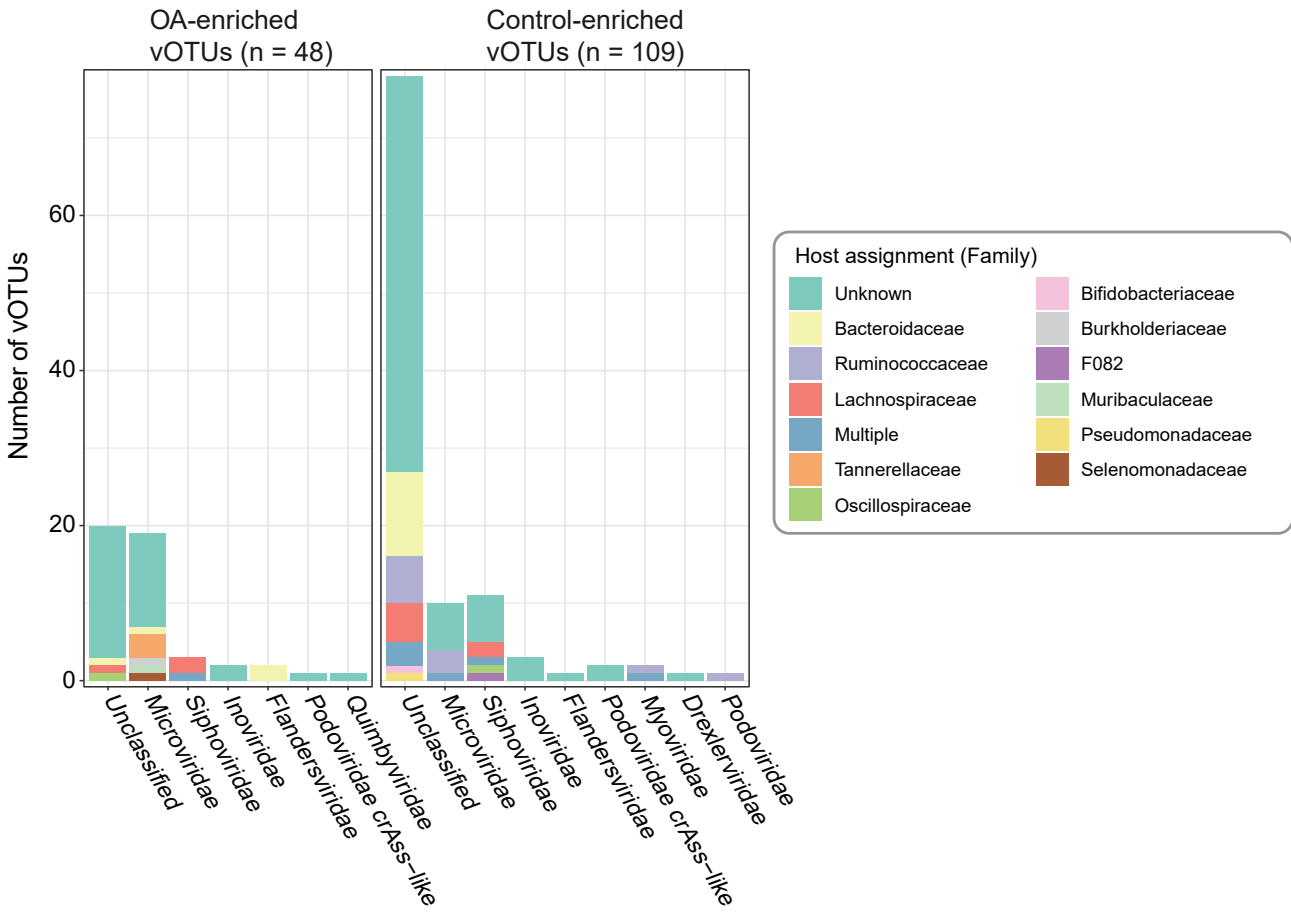

Supplement: Supplementary file 2 — Supplementary Material 2. [file 12967_2024_5374_MOESM2_ESM.pdf]

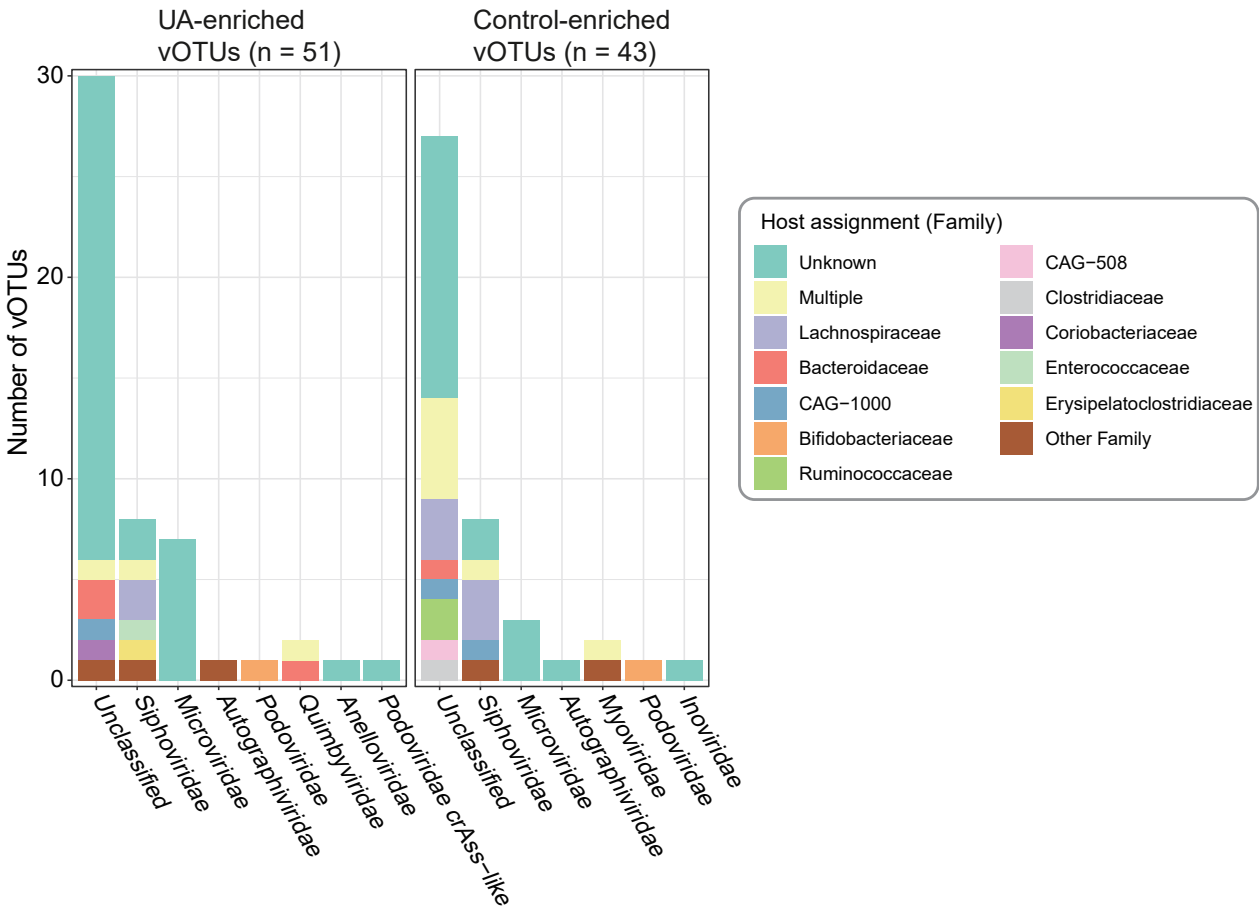

Supplement: Supplementary file 4 — Supplementary Material 4. [file 12967_2024_5374_MOESM4_ESM.pdf]
