# Supplementary material for: Distinct characteristics of the gut virome in patients with osteoarthritis and gouty arthritis
Source: J Transl Med. 2024 Jun 13;22:564. doi: 10.1186/s12967-024-05374-6 (PMC11170907; doi:10.1186/s12967-024-05374-6)
Supplement: Supplementary file 3 — Supplementary Material 3. [file 12967_2024_5374_MOESM3_ESM.pdf]

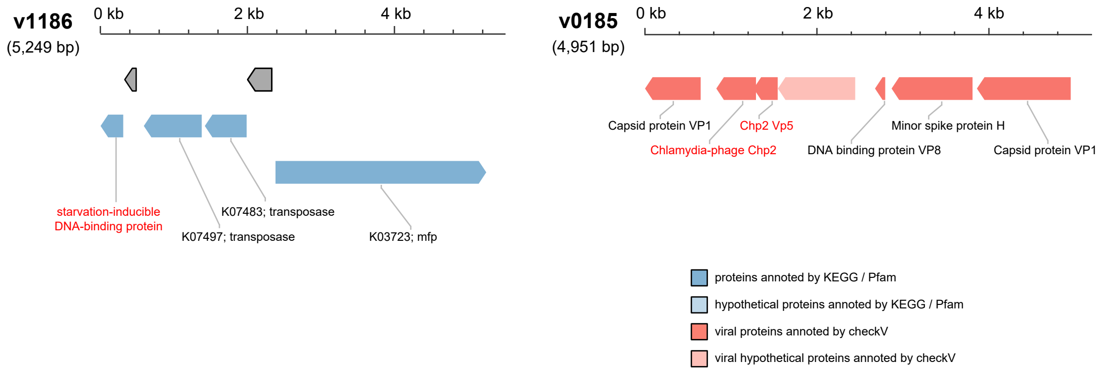
v1186
(5,249 bp)
0 kb
2 kb
4 kb
starvation-inducible DNA-binding protein
K07497; transposase
K07483; transposase
K03723; mfp
v0185
(4,951 bp)
0 kb
2 kb
4 kb
Capsid protein VP1
Chlamydia-phage Chp2
Chp2 Vp5
DNA binding protein VP8
Minor spike protein H
Capsid protein VP1
proteins annoted by KEGG / Pfam
hypothetical proteins annoted by KEGG / Pfam
viral proteins annoted by checkV
viral hypothetical proteins annoted by checkV
