# Supplementary material for: Distinct characteristics of the gut virome in patients with osteoarthritis and gouty arthritis
Source: J Transl Med. 2024 Jun 13;22:564. doi: 10.1186/s12967-024-05374-6 (PMC11170907; doi:10.1186/s12967-024-05374-6)
Supplement: Supplementary file 5 — Supplementary Material 5. [file 12967_2024_5374_MOESM5_ESM.pdf]

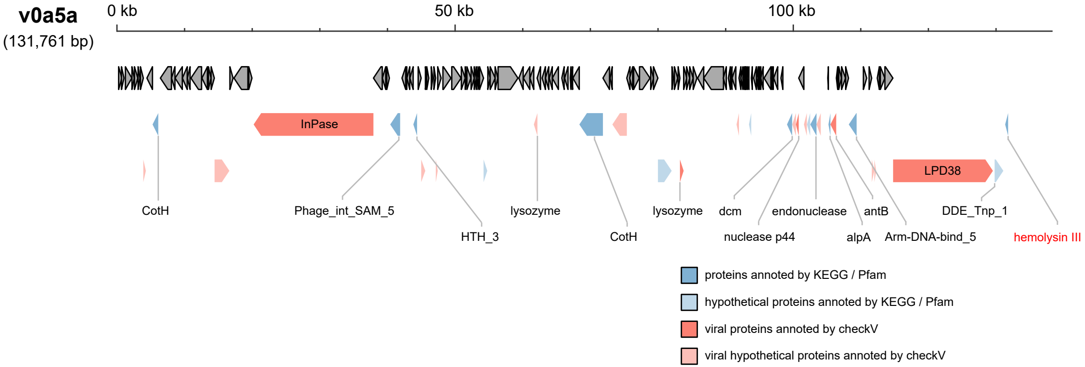

v0a5a
(131,761 bp)
0 kb
50 kb
100 kb
InPase
LPD38
CotH
Phage_int_SAM_5
HTH_3
lysozyme
CotH
lysozyme
dcm
nuclease p44
endonuclease
alpA
antB
Arm-DNA-bind_5
DDE_Tnp_1
hemolysin III
proteins annoted by KEGG / Pfam
hypothetical proteins annoted by KEGG / Pfam
viral proteins annoted by checkV
viral hypothetical proteins annoted by checkV
